# Different stimulation methods for the rat treadmill exercise model used in cardiovascular disease research

**DOI:** 10.3389/fphys.2026.1905960

**Published:** 2026-10-12

**Authors:** Yu Jiang, Lihua Fang, Jingwen Xiao, Qinfei Wei, Xiaona Lin, Yan Zhang

**Affiliations:** 1 Department of Cardiovascular Medicine, Fuzhou First General Hospital Affiliated with Fujian Medical University, Fuzhou, China; 2 Cardiovascular Disease Research Institute of Fuzhou City, Fuzhou, China; 3 The School of Clinical Medicine, Fujian Medical University, Fuzhou, China

**Keywords:** animal model, cardiovascular diseases, rats, stimulation method, treadmill training

## Abstract

**Objective:**

This paper aims to investigate the effectiveness of different stimulation methods on continuous treadmill exercise in rats, and to provide a rat treadmill exercise model used in cardiovascular disease research.

**Methods:**

Experimental rats were divided into 3 exercise intensity groups: low-intensity(L-EX), moderate-intensity(M-EX), and high-intensity(H-EX). Different stimulation methods (tactile, auditory, light, and electrical) were used to intervene when rats stopped during treadmill training. The number of successful stimulations was counted, and electrocardiograms (ECG) of the exercise process were recorded.

**Results:**

The success rate of both tactile stimulation and electrical stimulation was higher in the 3 different exercise intensity groups. The success rate of auditory stimulation was lower in the H-EX group than in the L-EX and M-EX groups. The success rate of light stimulation gradually decreased with the increase in exercise intensity. Overall, the other three stimulation methods were more successful than light stimulation. Furthermore, when electrical stimulation was used, ECG recordings showed marked baseline drift and waveform distortion caused by electrical artifacts, which made reliable interpretation of cardiac signals difficult.

**Conclusion:**

In constructing a rat treadmill exercise model for cardiovascular disease research, tactile stimulation appears preferable among the tested methods when the rat stops on the treadmill, as it effectively motivates continued running without interfering with ECG recordings.

## 1 Introduction

Cardiovascular Diseases (CVDs), including coronary heart disease, arrhythmia, and heart failure, are the leading cause of death worldwide. According to the World Health Organization (WHO), about 17.9 million people die of CVD every year, accounting for 32% of all global deaths (World Health Organization, 2024). Cardiovascular diseases not only cause great health damage to individual patients but also bring a heavy burden to the society and economy (Li et al., 2023). Therefore, an in-depth study of the pathogenesis of cardiovascular diseases and the search for feasible preventive and therapeutic methods are of great public health significance.

In recent years, exercise has come into view as an effective means that can prevent and treat CVD (Tucker et al., 2022). Exercise and CVD risk present a ‘dose-effect’ curve, in which moderate exercise improves cardiovascular function and reduces the risk of CVD, while excessive or inappropriate exercise may cause damage to the cardiovascular system (de Sousa et al., 2019; Perry et al., 2023).

Animal models are indispensable tools for in-depth study of disease mechanisms. Rats have become one of the preferred animal models in cardiovascular disease research due to their small size, rapid reproduction, easy feeding and management, and the similarity of the structure and function of their cardiovascular system to humans (Chen et al., 2022; Jin et al., 2024). Currently, the main feasible exercise modes for rats are running a table, swimming, rotating wheels, and climbing a ladder (Poole et al., 2020). Treadmill exercise is the most commonly used and effective training modality for the treatment and prevention of diseases due to its convenience, ease of operation, and precisely adjustable exercise speed, which can simulate the exercise state in the natural environment (Wang et al., 2020). Therefore, in order to further explore the role of exercise in affecting cardiovascular function and related molecular mechanisms, it is of great significance to construct a suitable and effective rat treadmill exercise model for cardiovascular disease research.

Most of the current running platforms are set up with 4 to 8 running tracks, which can meet the running requirements of animals of different sizes, and can be set with appropriate speed, duration, gradient, and intervention methods according to the experimental design (Li and Dong, 2023). To make the rats better complete the set ground running platform exercise program, the running platform mainly has electrical stimulation, auditory stimulation light stimulation, and other intervention modes. In our team’s previous experiments on running platform exercise, we found that rats not only stopped at exhaustion, but also stopped at times during the continuous running process before exhaustion, and the duration of the stopping time varied from a few seconds to up to 2 minutes, and some even did not move since then. At this point, the rats would resume movement when given an artificial stimulus intervention. This shows that running platform intervention is important for rat running exercise, and this paper investigates the effects of different Stimulation methods of the treadmill on the sustained exercise of rats, in order to supplement the application of the rat treadmill exercise model in the study of cardiovascular diseases. We hypothesized that non-noxious, contact-based stimulation (tactile) would offer the most favorable balance between a high success rate in motivating continued exercise and minimal interference with concurrent physiological recordings, such as the electrocardiogram (ECG).

## 2 Materials and methods

### 2.1 Experimental animals

24 healthy male Sprague-Dawley (SD) rats weighing 290.33 ± 5.92 g at 9 weeks of age. SI PEI FU (Beijing) Biotechnology Co. Ltd., Animal Production License No. SCXK (Beijing) 2024-0001, was the supplier of the SD rats, who were kept in individual cages with unrestricted access to food and water, a regular circadian rhythm, and a temperature of (23 ± 2)°C and relative humidity of 40%~60% in the animal room. The study protocol and experiments were conducted according to the guidelines of the Institutional Animal Care and Use Committee of Fujian Medical University and were approved by the Institutional Ethics Committee of Fujian Medical University(Approval No. IACUC FJMU 2025-Y-3074). All methods and results are reported in accordance with the ARRIVE 2.0 guidelines.

No animals or data points were excluded. No adverse events occurred, and no animals reached exhaustion; humane endpoints were defined as persistent immobility, vocalization, or signs of distress, and no animal met these criteria. Only male rats were used to avoid confounding effects of the estrous cycle on exercise performance and cardiac parameters. At the end of the experiment, rats were euthanized under anesthesia.

### 2.2 Treadmill training

The treadmill, model ZH-PT/5S was purchased from Anhui Zhenghua Biological Instrument Co., Ltd (Huaibei, Anhui, China), as shown in **Figure 1A**. TT was all performed at night in dimly lit ambient conditions with a temperature of (23 ± 2)°C and relative humidity of 40% to 60%.

**Figure 1:**
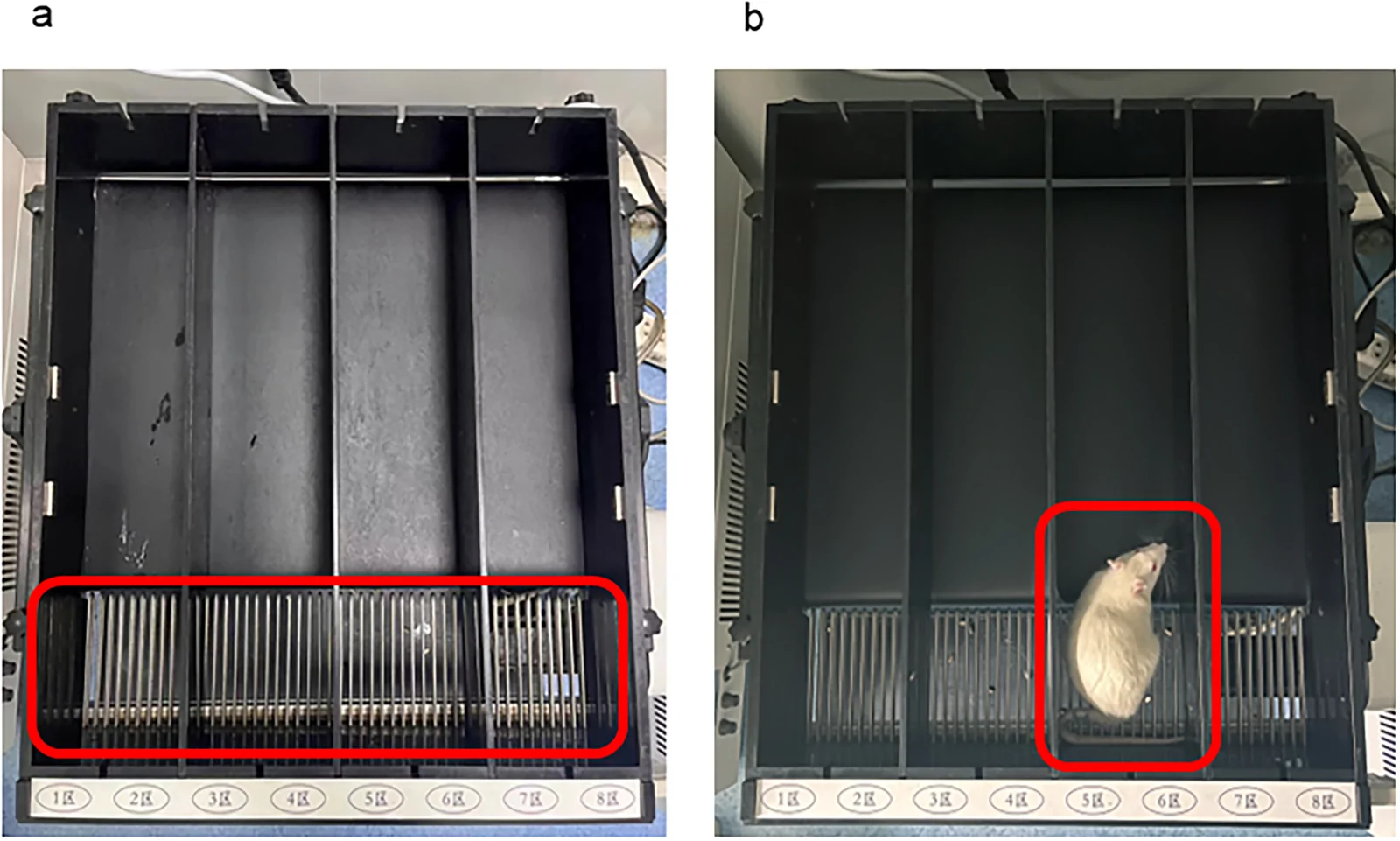
Rat treadmill and rat exhaustion status picture. The electrical stimulation area is indicated by the red-circled portion of the rat running platform in **(A)**. The rat running exhaustion state graphic is displayed in **(B)**.

Training for acclimatization: Before the official trial, all rats had a week of acclimatization training (exercise time of 10 minutes, once daily, at a speed of 8 m/min) to acclimate them to the running platform setting.

Formal experiment: 24 rats were randomly assigned to 3 groups: 8 rats in each group were assigned to low-intensity exercise group (L-EX), moderate-intensity exercise group (M-EX), and high-intensity exercise group (H-EX).

The following treadmill exercise routines were created for the various exercise intensities in this trial based on the literature review and the team’s prior experience (Guo et al., 2020): Twelve meters per minute was the speed at which the low-intensity exercise group (L-EX), sixteen meters per minute was the speed of the moderate-intensity exercise group (M-EX), and twenty-two meters per minute was the speed of the high-intensity exercise group (H-EX). Each session was fixed at 30 minutes; no animal reached exhaustion within this period. Rats had to shift from stomping to a crouching position, have their abdomens touch the running track surface, stay at the end of the track and be unable to run any farther, and be unable to be motivated to run by any stimulus to be considered exhausted (Qiao et al., 2012). as seen in **Figure 1B**.

### 2.3 Stimulation methods

The following stimulations were used:

(1) Tactile stimulation: A standardized wooden stick was used to gently touch the rat’s tail. All tactile stimulations were performed by the same experimenter to maintain consistency across trials. Each contact lasted approximately 1 second and was defined as a gentle touch without pressure that did not cause vocalization or persistent avoidance. (2) Auditory stimulation: A plastic film (20 cm×10 cm) was rubbed at a constant frequency at a distance of approximately 10 cm from the rat. The same type of film and the same experimenter were used throughout. (3) Light stimulation: The built in light source of the treadmill was used to continuously illuminate the rat’s tail area. (4) Electrical stimulation: Electrical pulse from the treadmill device (2 mA constant current). The intensity was pre-validated as the minimum effective stimulus to elicit a response without causing avoidable distress.

Timing of stimulation: the rat remains at the end of the runway for more than 2 seconds after stopping its limb movements and automatically traveling backward with the runway. Stimulation success criterion: the rat advances again within 2 seconds of the 4 stimuli mentioned above. The rat will be moved to the front end of the runway so that it can move again if it takes more than 2 seconds, which is considered a failure of the intervention. Success rate was defined as the number of successful stimulations divided by the total number of stimulation attempts for each rat, under each stimulation method, on each experimental day. The first stopping time was defined as the time from the start of a single session to the first stopping event, and total stopping time was defined as the cumulative duration of all stopping events during a single session. Both were recorded manually by the same experimenter using a timing device.

This was a balanced rotation design in which each 4-day block contained all four stimulation methods, and the rotation was repeated across three cycles, ensuring that each method appeared equally often in each temporal position across animals. Rats were randomly assigned tail numbers NO.1 to NO.8 using a random number table. The starting modality was determined by tail number: NO.1–2 began with tactile, NO.3–4 with auditory, NO.5–6 with light, and NO.7–8 with electrical stimulation. The detailed sequence is illustrated in **Figure 2**.

**Figure 2:**
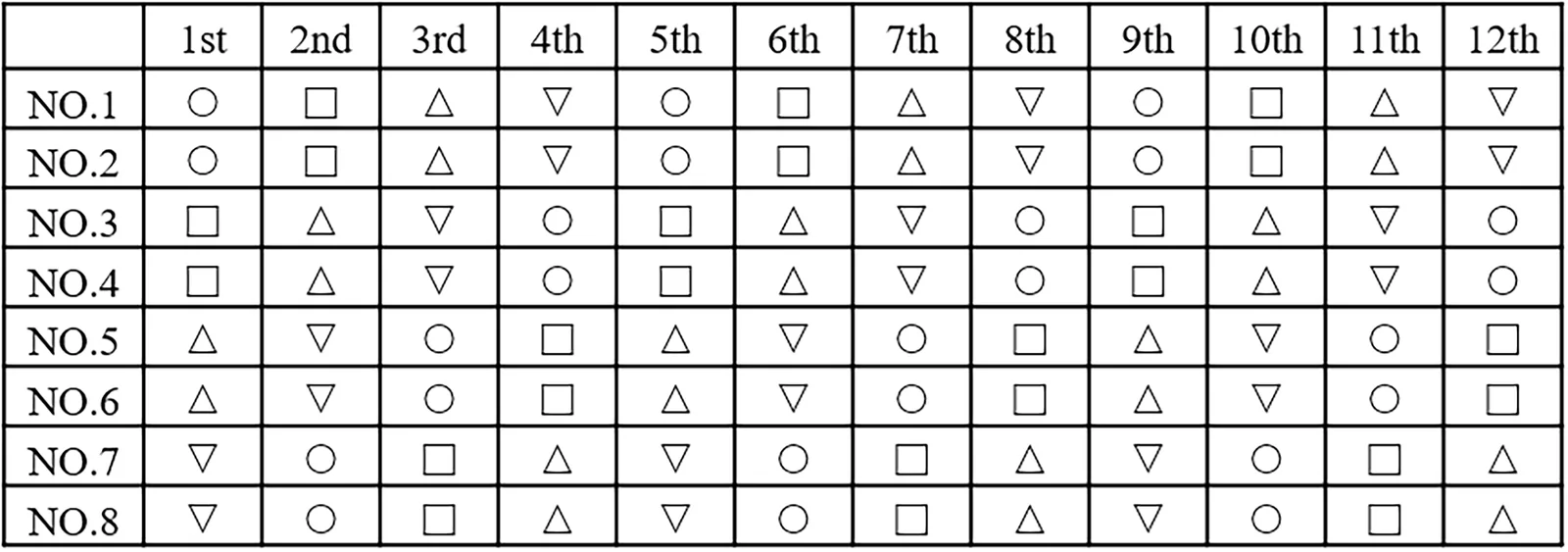
Specific experimental setups of different Stimulation methods for rat treadmill training. 1st to 12th represent the first day to the 12th day of the experiment, respectively. NO.1 to NO.8 represent the number of 8 rats in each group, respectively. The symbols ○, □, △, and ▽ stand for tactile, auditory, light, and electrical stimulation, respectively.

### 2.4 ECG recording while exercising

A rechargeable animal telemetry device (model KLR-2, acquired from Shanghai Kexin Medical Biotechnology Company, Shanghai, China) was used to record the ECG data of the rat treadmill exercise model. Using software from the same business, the ECG data were then monitored in real-time during exercise after the electrodes were implanted under gas anesthesia into the subcutaneous region of the rat’s left upper limb and right lower leg. Peri-operative analgesia and antibiotics were administered for telemetry implantation, with a 1-day recovery period. The ECG findings are presented as qualitative evidence of signal interference caused by electrical artifacts. No claims regarding physiological or autonomic effects are made.

### 2.5 Statistical approach

The sample size was calculated using the resource equation E=N-K=Kn-K=K(n-1). Here, N represents the total number of animals, n is the sample size per group, K represents the number of treatment groups, and E is the error degrees of freedom. This experiment used three groups of rats with different exercise intensities. Based on preliminary experimental data, the effect size and variance were estimated. With K = 3 groups and n = 8 rats per group, N = 24 and E = 3×(8−1) = 21. According to the resource equation method (Arifin and Zahiruddin, 2017), the acceptable range for E is 10–20; however, this range is described as a reference guideline rather than a strict cutoff. We retained 8 rats per group for the following reasons: (1) the additional rat per group (compared with n = 7, which would yield E = 18) provided a margin for potential animal loss or data exclusion during the 12-day experimental protocol; (2) the slightly higher E value (21 vs. 20) does not compromise statistical validity, as the resource equation method is a guide rather than a strict cutoff; and (3) the 3Rs principle was respected by not increasing the sample size beyond what was necessary. Following review and confirmation by a biostatistics expert, a total of 24 rats were ultimately selected, with 8 rats in each group. This was an exploratory study with a limited between-group sample size (n = 8 per intensity condition).

Statistical analyses were performed using SPSS 26.0. Normality of residuals was assessed using the Shapiro–Wilk test, homogeneity of variance using Levene’s test, and sphericity using Mauchly’s test. When sphericity was violated, the Greenhouse–Geisser correction was applied. To compare the success rates of different stimulation methods across exercise intensity groups and to account for potential order effects, a three-way mixed-design repeated-measures analysis of variance (ANOVA) was performed, with Stimulation Method (tactile, auditory, light, electrical) and Cycle (1st, 2nd, and 3rd rounds of stimulation rotation) as within-subject factors, and Exercise Intensity Group (L-EX, M-EX, H-EX) as the between-subject factor. Simple main effects analysis was conducted to decompose significant interactions. For all pairwise *post-hoc* comparisons, Sidak’s multiple comparison test was applied to control for Type I error. Effect sizes (partial η^2^) and 95% confidence intervals are reported for main effects, interactions, and key pairwise comparisons. The first cycle (Days 1–4) and the last cycle (Days 9–12) were used as the pre- and post-intervention periods. Paired-samples t-tests were used to compare the frequency of stops and the overall stimulation success rate between the first cycle and the third cycle. One-way ANOVA was used to compare baseline exercise characteristics (first stop time, total stop time) among the three exercise intensity groups, with Sidak correction applied for multiple comparisons. A sensitivity analysis using arcsine square root transformed success rates was performed to assess the robustness of the findings. A significance level of p < 0.05 was considered statistically significant for all analyses.

## 3 Results

### 3.1 Comparison of the basal condition of exercise in rats

During the 30-minute treadmill exercise, none of the rats in the 3 groups experienced exhaustion. The H-EX group experienced the first stop point earlier than the L-EX group, and the difference was statistically significant (*p* = 0.003). As shown in **Figure 3A**. The total stop time during exercise increased as the intensity of activity increased, and the total stop time during exercise was compared two-by-two between the 3 groups (*p* < 0.001). As seen in **Figure 3B**. Please refer to **Supplementary Table S1** for the complete data.

**Figure 3:**
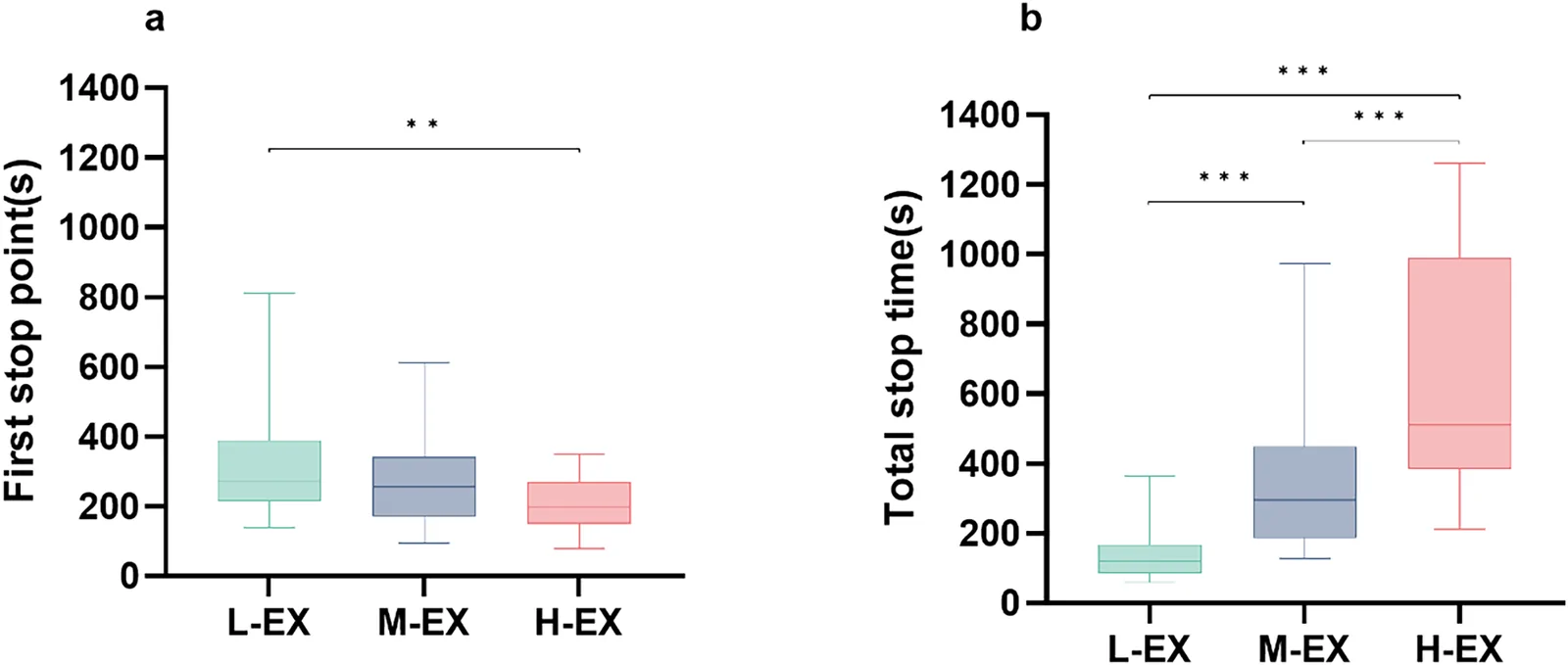
Comparison of the basal condition of exercise in rats. **(A)** Represents the first stop point during exercise for different exercise intensity groups. Panel **(B)** indicates the total stop time during exercise for different exercise intensity groups. L-EX indicates the low-intensity exercise group. M-EX indicates the moderate-intensity exercise group. H-EX indicates the high-intensity exercise group. ** denotes *p* < 0.01. *** denotes *p* < 0.001.

### 3.2 Comparison of the success rates of different stimulation methods

A three-way mixed-design repeated-measures ANOVA was performed with Stimulation Method and Cycle as within-subject factors and Exercise Intensity Group as the between-subject factor.

The analysis revealed a significant main effect of Stimulation Method [F(1.702, 35.739) = 1363.773, *p* < 0.001, partial η^2^ = 0.985], a significant main effect of Exercise Intensity Group [F(2, 21) = 9.319, *p* = 0.001, partial η^2^ = 0.470], and a significant Stimulation Method × Exercise Intensity Group interaction [F(3.404, 35.739) = 3.634, *p* = 0.018, partial η^2^ = 0.256]. The Stimulation Method × Cycle interaction was also significant [F(2.225, 46.723) = 4.676, *p* = 0.012, partial η^2^ = 0.182], but *post-hoc* inspection confirmed that the relative ranking of the four methods remained stable across cycles. Full results are provided in **Supplementary Table S2**. A sensitivity analysis using arcsine square root transformed data, presented in **Supplementary Table S3**, yielded consistent results for the main effect of Stimulation Method, confirming the robustness of the findings.

Pairwise comparisons (Sidak-corrected) showed that tactile stimulation (0.970 ± 0.005) and electrical stimulation (0.961 ± 0.005) achieved the highest success rates with no significant difference between them (mean difference = 0.009, 95% CI: −0.007 to 0.025, p = 0.502). Auditory stimulation (0.940 ± 0.010) was significantly lower than tactile stimulation (mean difference = 0.030, 95% CI: 0.001 to 0.058, p = 0.037) but comparable to electrical stimulation (mean difference = −0.021, 95% CI: −0.049 to 0.007, p = 0.236). Light stimulation (0.259 ± 0.014) was significantly inferior to all other methods (all p < 0.001; e.g., vs. tactile: mean difference = 0.711, 95% CI: 0.662 to 0.760; vs. electrical: mean difference = −0.702, 95% CI: −0.747 to −0.657).For complete statistical data, reference **Supplementary Tables S4** and **S5**.

Simple main effects analysis (Sidak-corrected) further revealed that tactile and electrical stimulation maintained consistently high success rates across all three intensity groups, with no significant differences between groups for either method (*p*> 0.05), as illustrated in **Figures 4A, D**. In contrast, auditory stimulation showed a significant decline in the H-EX group compared to the L-EX and M-EX groups (*p* < 0.01 and *p* < 0.001, respectively), as seen in **Figure 4B**. Light stimulation exhibited a progressive decline with increasing exercise intensity, with significant differences between all three groups (*p* < 0.05), as indicated in **Figure 4C**.

**Figure 4:**
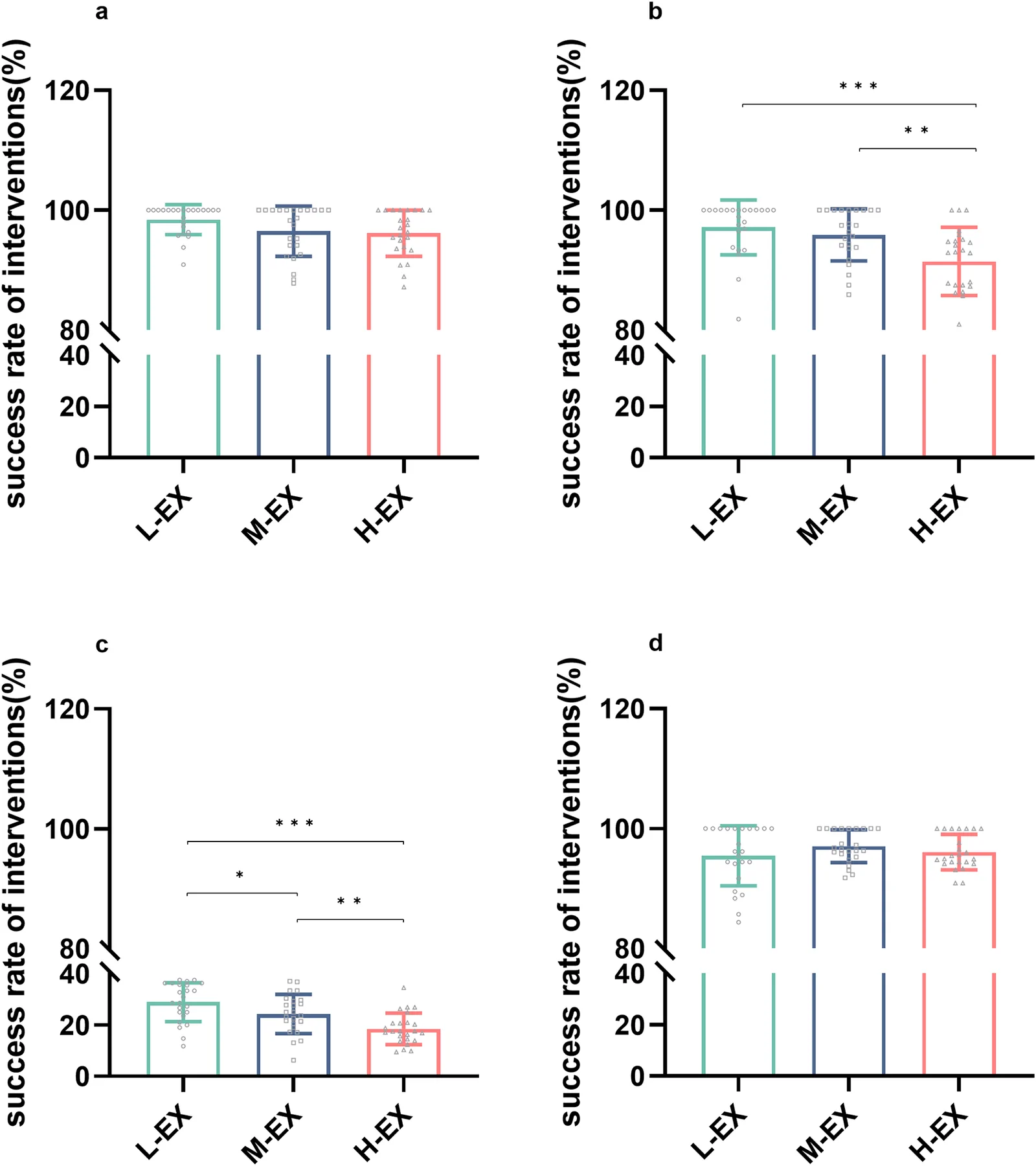
Comparison of the success rate of a particular stimulation method in different exercise intensity groups. **(A)** Represents the ratio of the success rate of tactile stimulation during exercise for different exercise intensity groups. **(B)** Represents the comparison of the success rate of auditory stimulation during exercise for different exercise intensity groups. **(C)** Represents the comparison of the success rate of light stimulation during exercise in different exercise intensity groups. **(D)** Represents the comparison of the success rate of electrical stimulation during exercise in different exercise intensity groups. L-EX indicates the low-intensity exercise group. M-EX indicates the moderate-intensity exercise group. H-EX indicates the high-intensity exercise group. * denotes p<0.05.** denotes *p* < 0.01. *** denotes *p* < 0.001.

Within each intensity group, the patterns were consistent: in the L-EX group, tactile stimulation outperformed electrical stimulation (*p* = 0.035); in the M-EX group, no significant differences were observed among tactile, auditory, and electrical stimulation; in the H-EX group, tactile and electrical stimulation were comparable but both outperformed auditory stimulation (*p* < 0.01). In all three groups, light stimulation was significantly inferior to the other three methods (*p* < 0.001). Overall, the ranking of success rates was consistent across all conditions: tactile and electrical stimulation were comparably effective, both superior to auditory stimulation, with light stimulation being the least effective.

### 3.3 Comparison of related conditions between the early and late cycles

The frequency of stops decreased significantly from the first cycle (Days 1–4) to the third cycle (Days 9–12) (mean difference = −27.83 ± 40.72, *p* = 0.003), as shown in **Figure 5A**. Despite a modest increase in the overall stimulation success rate, this change did not reach statistical significance (mean difference = 2.13 ± 7.68, *p*>0.05), as seen in **Figure 5B**. See **Supplementary Table S6** for the data.

**Figure 5:**
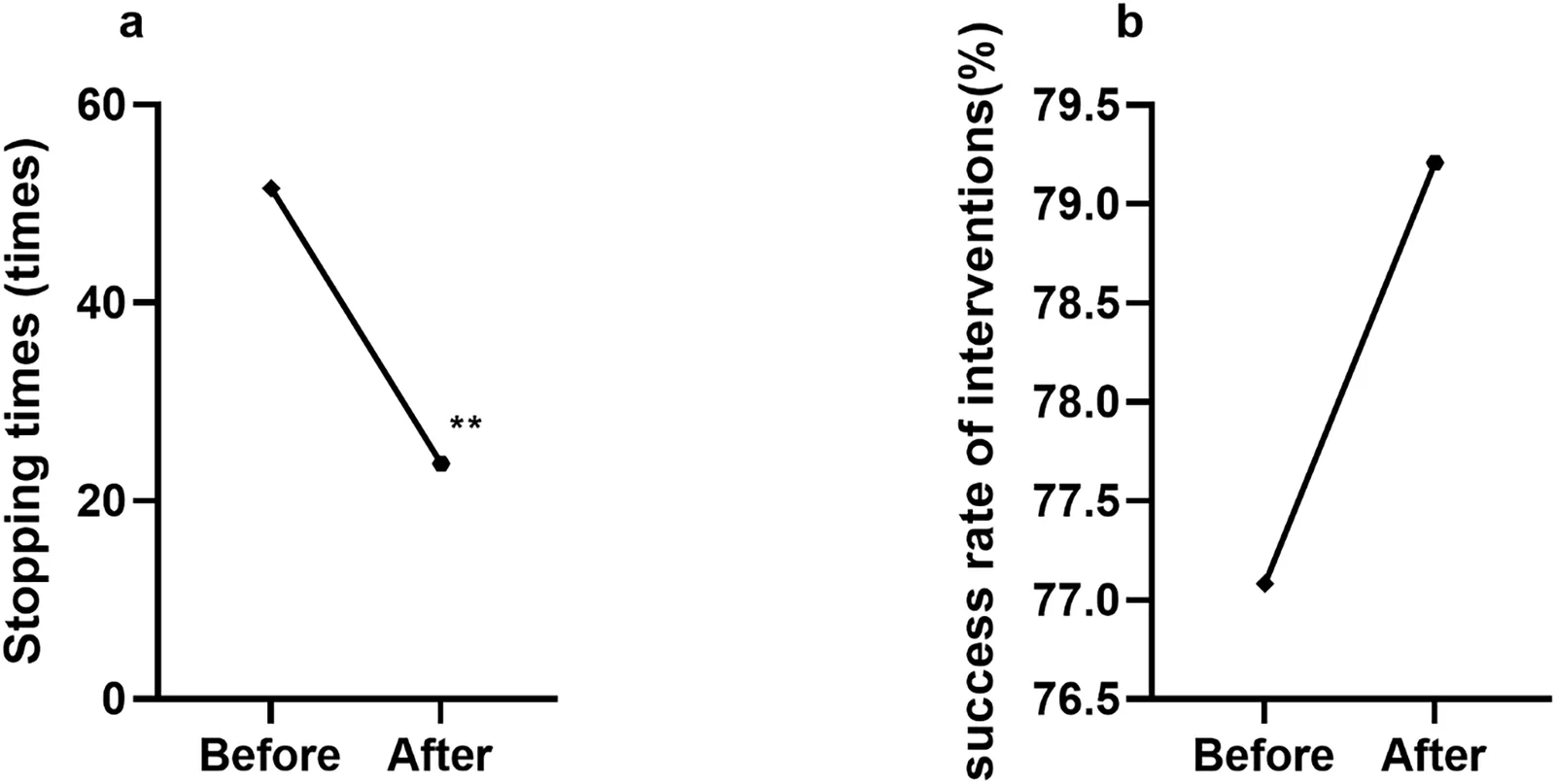
Comparison of related conditions between the first cycle (Days 1–4) and the third cycle (Days 9–12). **(A)** Comparison of the number of stops between the two cycles. **(B)** Comparison of the overall stimulation success rate between the two cycles. ** denotes *p* < 0.01.

### 3.4 Qualitative visual assessment of ECG recordings during exercise

**Figure 6A** shows a rat exercising on the treadmill with the ECG telemetry device. **Figure 6B** displays representative ECG recordings during normal exercise without any stimulation, showing a stable baseline and clear QRS complexes. As shown in **Figures 6C–E**, tactile, auditory, and light stimulation caused minimal interference with ECG recordings, with stable baselines and clearly discernible waveforms. In marked contrast, electrical stimulation severely disrupted ECG acquisition, inducing substantial baseline drift and high-frequency noise that obscured the QRS complexes (**Figures 6F, G**).

**Figure 6:**
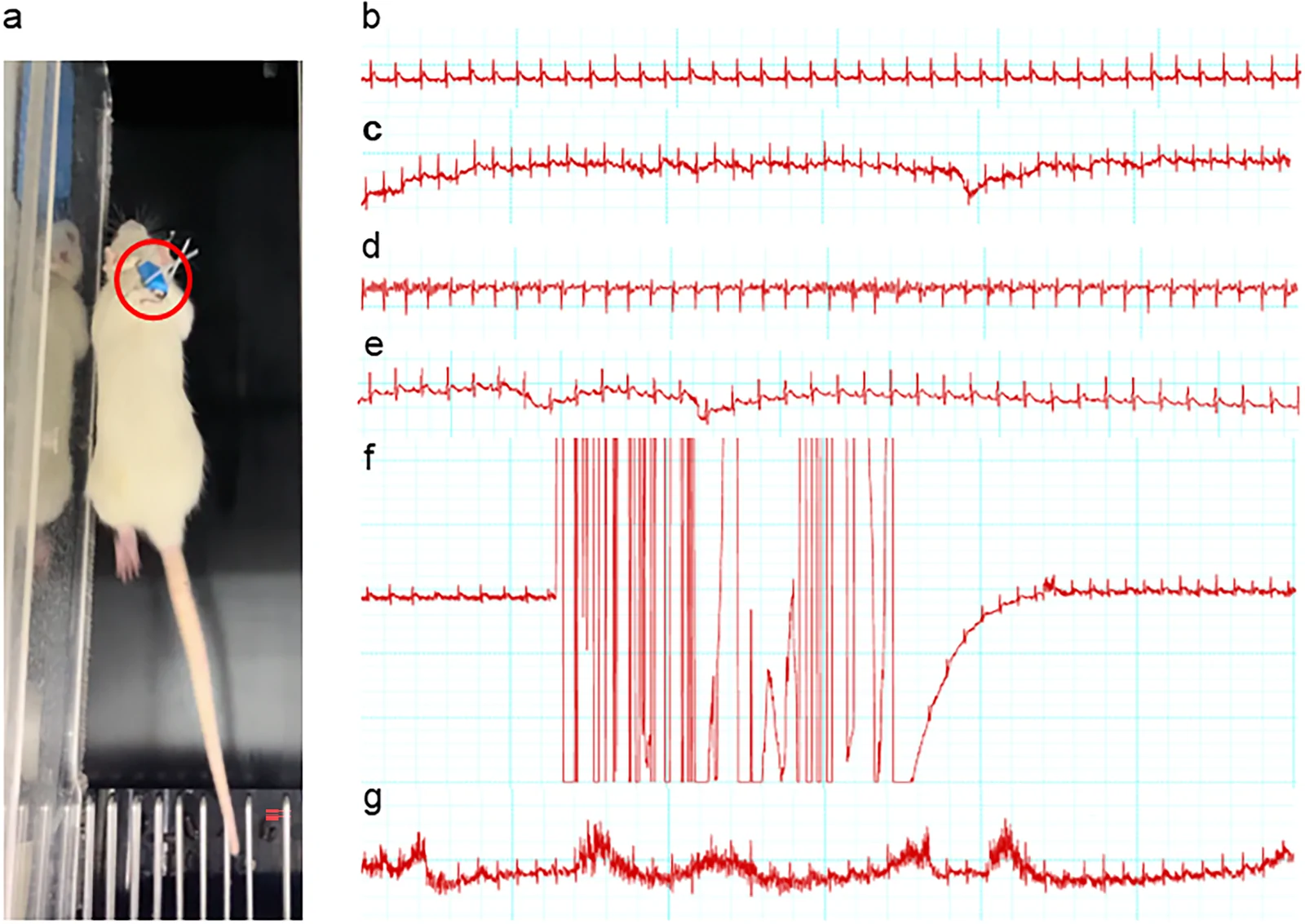
Qualitative visual assessment of ECG recordings during exercise. **(A)** A rat exercising on the treadmill with the ECG telemetry device. **(B)** Representative ECG recording during normal exercise without stimulation, showing a stable baseline and clear QRS complexes. **(C–E)** ECG recordings during tactile, auditory, and light stimulation, respectively, showing minimal interference with stable baselines and clearly discernible waveforms. **(F, G)** ECG recordings during electrical stimulation, showing substantial baseline drift and high-frequency noise that obscured the QRS complexes.

## 4 Discussion

The results of this study show that during treadmill exercise in rats, the success rates of tactile stimulation, electrical stimulation, and auditory stimulation were all significantly higher than that of light stimulation. In addition, during exercise, stimulation methods such as tactile stimulation, auditory stimulation, and light stimulation interfere less with ECG recordings, whereas electrical stimulation, for example, seriously affects ECG recordings. In constructing a rat treadmill exercise model for cardiovascular disease research, tactile stimulation may be the relatively optimal stimulation when the rat stops at the treadmill to not interfere with ECG recordings.

TT is a method used to train and assess the physical fitness of small animals, such as rats and mice (Fujimaki et al., 2021). It is conducted using a treadmill with adjustable speed, and the surface texture of the treadmill is designed to help the animals maintain grip, ensuring stability while moving. During the experiment, animals are motivated to continue running through physical contact and stimuli from the treadmill apparatus (such as electric shocks, sounds, and lights). If the animal’s speed falls below the set value, the stimulation device is triggered to prompt it to return to the set speed.

Exercise can both be a trigger for certain cardiovascular diseases and serve as a therapeutic measure for some of them (Valenzuela et al., 2023). Different intensities of exercise have varying effects on the heart. For instance, the relationship between exercise intensity and atrial fibrillation follows a J-shaped or U-shaped dose-response curve (Jin et al., 2019). This may be related to mitochondrial function, oxidative stress, and lipid metabolism (Valenzuela et al., 2023). Therefore, establishing a cardiovascular rat model for exercise is of significant importance for further research into the impact of exercise on cardiovascular diseases.

The results of this paper showed that the intervention success rates of tactile stimulation and acoustic stimulation were similar in the L-EX and M-EX groups, and the differences were not statistically significant; therefore, we suggest that when constructing a low- to moderate-intensity exercise model for rats used in cardiovascular disease research, we can use either tactile stimulation or acoustic stimulation interventions to make the rats exercise continuously. Currently, there are treadmill devices equipped with airflow jets, which may be an intervention that simulates limb contact combined with light acoustic stimulation.

Light stimulation, as a non-contact stimulation method, usually does not interfere with the collection of physiological data, but the results of the present study showed that light stimulation was ineffective, which may be due to the inability of light stimulation to give the rats sufficient motivation. The experimental results also showed that the success rate of light stimulation decreased as the intensity of exercise increased, which may indicate that light stimulation as a stimulation modality was unable to overcome the fatigue produced by high-intensity training in rats. Some studies have shown that glutamine has the effect of relieving exercise fatigue and reducing the degree of oxidative stress in skeletal muscle in rats (Jiang et al., 2021), which shows that the physiological state of rats may also be an influencing factor, and when the rats are in a state of discomfort, the skeletal muscle may not be able to respond to light stimulation, a relatively weak stimulation method.

Electrical stimulation was more effective, but during our experiments, some rats remained in the electrically stimulated area and chose not to move even after continuous electrical stimulation was given again, which may be related to the development of tolerance in the rat, or it may be related to the fact that it has found a certain posture that is not stimulated because after moving the rat’s calf, it immediately felt the electric current and thus continued to move forward.

We believe that when the rat treadmill exercise model is used for cardiovascular disease research, it is important to record data about the ECG signal, which is not only limited to the comparison of the ECG data before and after the exercise, but also the recording of the heart rate changes during the exercise, the occurrence of arrhythmic events, and the variability of the heart rate (Tomsits et al., 2022). The fourth part of the results showed that using electrical stimulation to intervene in exercise, the baseline of the ECG data from rats was extremely unstable, and further data analysis could not be performed according to normal procedures. Therefore, electrical stimulation is not suitable as an exercise stimulation modality for the rat treadmill exercise model that requires the recording of ECG signals. An earlier study has shown that electrical stimulation affects glucose metabolism to a certain extent by affecting blood glucose changes more significantly than tactile stimulation during treadmill exercise in rats (Xiao and Guo, 1998). In addition, it has been shown that low-frequency electrical stimulation induces skeletal muscle CAIII expression, which in turn fatigues the skeletal muscle (Shang et al., 2009). Although the use of tactile stimulation is time-consuming and labor-intensive compared to electrical stimulation, it is a better intervention for sustained exercise in the rat treadmill exercise model used for cardiovascular disease research.

Although different stimulation methods may in principle modulate autonomic nervous system activity, the present study did not directly assess autonomic function. Therefore, the choice of stimulation method should take into account its potential impact on the autonomic nervous system, especially in studies investigating cardiac electrophysiology, arrhythmias, or autonomic function. In the present study, our ECG recordings demonstrated that electrical stimulation produced substantial artifacts that precluded reliable interpretation of cardiac signals. These ECG findings are presented as qualitative evidence of signal interference caused by electrical artifacts, and no claims regarding physiological or autonomic effects are made. Exercise intensity and modality can influence systemic physiological and redox-related responses in humans (Soslu et al., 2023; Uysal and Soslu, 2026), but these findings should not be extrapolated directly to rat treadmill stimulation or ECG artifact. Future studies employing non-electrical stimulation methods or effective artifact-filtering techniques are warranted to quantitatively evaluate the autonomic effects of different stimulation modalities during treadmill exercise using Heart rate variability (HRV) analysis (Peltola, 2012).

After three cycles of stimulation intervention, the number of stops during exercise in the third cycle was significantly reduced compared with the first cycle, suggesting that effective intervention stimulation leads to a better adaptation to platform running exercise. In addition, the appropriate adaptation training, the environment of the exercise, and the design of the experiment may affect the effectiveness of the stimulation modality, especially the ambient temperature (Andrade et al., 2023). This experiment was designed to ensure the comparability between groups in order to find out the most appropriate stimulation modality.

This experiment shows the comparative results of different stimulation methods on the sustained locomotor activity of rats, which is statistically significant, but of course, there are individual differences in rats, so we suggest that different rats can try individualized stimulation methods in pre-tests, and can also be intervened with a combination of different stimulation methods.

In this experiment, we investigated the efficiency of different stimulation methods on sustained exercise in rats, which provides data and theoretical support for further construction of a rat treadmill exercise model applied to cardiovascular diseases. However, this study has several limitations. First, only rats were used, and mice were not included. Second, all animals were healthy, and treadmill experiments were not performed on rats with pre-existing cardiovascular disease, as our initial plan was to conduct cardiovascular disease modeling or cardiac electrophysiological examinations after the exercise period. Future studies will extend these findings to mice and to animal models of coronary heart disease and arrhythmia.

## 5 Conclusion

This study provides methodological evidence for selecting stimulation methods in rat treadmill exercise models used in cardiovascular disease research. Among the tested methods, tactile stimulation appears preferable for maintaining continuous treadmill running while preserving ECG readability, as it effectively motivated rats to resume running without interfering with ECG recordings.

## Data Availability

The original contributions presented in the study are included in the article/**Supplementary Material**, further inquiries can be directed to the corresponding author/s.
