# Supplementary material for: Different stimulation methods for the rat treadmill exercise model used in cardiovascular disease research
Source: Front Physiol. 2026 Oct 12;17:1905960. doi: 10.3389/fphys.2026.1905960 (PMC13696206; doi:10.3389/fphys.2026.1905960)
Supplement: Supplementary file 1 [file DataSheet1.pdf]

**Supplementary Material**

**Supplementary Table S1 Comparison of the basal condition of exercise in rats.**

| Items | L-EX | M-EX | H-EX | *P* | PC | *P* |
|---|---|---|---|---|---|---|
| First stop point | 270.00 (214.50,389.00) | 255.50 (171.00,341.50) | 196.00 (149.50,269.50) | 0.012 | L-EX vs M-EX | 0.205 |
| | | | | | L-EX vs H-EX | 0.003 |
| | | | | | M-EX vs H-EX | 0.101 |
| Total stop time | 120.00 (85.50,166.00) | 295.50 (186.25,448.75) | 510.50 (385.50,988.25) | < 0.001 | L-EX vs M-EX | < 0.001 |
| | | | | | L-EX vs H-EX | < 0.001 |
| | | | | | M-EX vs H-EX | < 0.001 |

Note: L-EX: low-intensity exercise group; M-EX: moderate-intensity exercise group; H-EX: high-intensity exercise group; PC: Pairwise Comparisons.

**Supplementary Table S2 Repeated-measures ANOVA results for the original success-rate data**

| Source | *F* | df1 | df2 | *P* | Partial $\eta^2$ |
|---|---|---|---|---|---|
| **Within-subjects effects** | | | | | |
| Stimulation Method | 1363.773 | 1.702 | 35.739 | < 0.001 | 0.985 |
| Stimulation Method × Exercise Intensity Group | 3.634 | 3.404 | 35.739 | 0.018 | 0.256 |
| Cycle | 3.114 | 1.438 | 30.207 | 0.074 | 0.129 |
| Cycle × Exercise Intensity Group | 0.926 | 2.877 | 30.207 | 0.437 | 0.082 |
| Stimulation Method × Cycle | 4.676 | 2.225 | 46.723 | 0.012 | 0.182 |
| Stimulation Method × Cycle × Exercise Intensity Group | 3.656 | 4.450 | 46.723 | 0.009 | 0.258 |
| **Between-subjects effect** | | | | | |
| Exercise Intensity Group | 9.319 | 2 | 21 | 0.001 | 0.470 |

Note: Data were analyzed using a three-way mixed-design repeated-measures ANOVA. Mauchly's test indicated that the sphericity assumption was violated for Stimulation Method ($p < .001$), Stimulation Method × Exercise Intensity Group ($p < .001$), and Stimulation Method × Cycle ($p < .001$); therefore, the Greenhouse–Geisser correction was applied to all within-subject effects. Sidak correction was applied for pairwise comparisons. Partial $\eta^2$ = SS_effect / (SS_effect + SS_error).

**Supplementary Table S3 Sensitivity analysis using arcsine square root transformed success rates**

| Source | *F* | df1 | df2 | *P* | Partial $\eta^2$ |
|---|---|---|---|---|---|
| **Within-subjects effects** | | | | | |
| Stimulation Method | 582.124 | 3 | 63 | < 0.001 | 0.965 |
| Stimulation Method × Exercise Intensity Group | 1.809 | 6 | 63 | 0.112 | 0.147 |
| Cycle | 6.693 | 2 | 42 | 0.003 | 0.242 |
| Cycle × Exercise Intensity Group | 0.801 | 4 | 42 | 0.531 | 0.071 |
| Stimulation Method × Cycle | 3.876 | 3.838 | 80.599 | 0.007 | 0.156 |
| Stimulation Method × Cycle × Exercise Intensity Group | 2.769 | 7.679 | 80.599 | 0.010 | 0.209 |
| **Between-subjects effect** | | | | | |
| Exercise Intensity Group | 6.090 | 2 | 21 | 0.008 | 0.367 |

Note: Data were analyzed using a three-way mixed-design repeated-measures ANOVA after arcsine square root transformation. Mauchly's test indicated that the sphericity assumption was met for Stimulation Method ($p = 0.057$) and Cycle ($p = 0.263$), but was violated for the Stimulation Method × Cycle interaction ($p = 0.024$); therefore, the Greenhouse–Geisser correction ($\varepsilon = 0.640$) was applied to that interaction. Sidak correction was applied for pairwise comparisons.The pattern of results was consistent with the primary analysis for the main effect of Stimulation Method.

**Supplementary Table S4 Success rates of the four stimulation methods across different exercise intensity groups**

| Success Rate (%) | L-EX | M-EX | H-EX |
|---|---|---|---|
| Tactile Stimulation | 98.42 | 96.48 | 96.16 |
| Auditory Stimulation | 97.13 | 95.87 | 91.44 |
| Light Stimulation | 28.92 | 24.29 | 18.50 |
| Electrical Stimulation | 95.46 | 97.06 | 96.07 |

Note: L-EX: low-intensity exercise group; M-EX: moderate-intensity exercise group; H-EX: high-intensity exercise group.

**Supplementary Table S5 Pairwise comparisons among the four stimulation methods (Sidak-corrected)**

| Comparison | Mean Difference | SE | *P* | 95% CI Lower | 95% CI Upper |
|---|---|---|---|---|---|
| Tactile vs. Auditory | 0.030 | 0.010 | 0.037 | 0.001 | 0.058 |
| Tactile vs. Light | 0.711 | 0.017 | < 0.001 | 0.662 | 0.760 |
| Tactile vs. Electrical | 0.009 | 0.005 | 0.502 | -0.007 | 0.025 |
| Auditory vs. Light | 0.681 | 0.018 | < 0.001 | 0.629 | 0.733 |
| Auditory vs. Electrical | -0.021 | 0.010 | 0.236 | -0.049 | 0.007 |
| Light vs. Electrical | -0.702 | 0.016 | < 0.001 | -0.747 | -0.657 |

**Supplementary Table S6 Comparison of related conditions between the first cycle and the third cycle**

| Variable | Comparison | Mean difference ± SD | *P* |
|---|---|---|---|
| Stopping times | Third cycle − First cycle | −27.83 ± 40.72 | 0.003 |
| Success rate of interventions | Third cycle − First cycle | 2.13 ± 7.68 | 0.188 |

Note: The data were analyzed using a paired t-test.
